# Is the coastal future green, grey or hybrid? Diverse perspectives on coastal flood risk management and adaptation in the UK

**DOI:** 10.1017/cft.2024.4

**Published:** 2024-02-12

**Authors:** Elina Apine, Tim Stojanovic

**Affiliations:** Marine and Coastal Environment Team, School of Geography & Sustainable Development, University of St Andrews, St Andrews, UK

**Keywords:** coastal change, managed realignment, coastal adaptation, sea-level rise, coastal protection

## Abstract

Climate change-induced sea level rise has exacerbated coastal change putting millions of people at risk from coastal hazards, such as flooding and coastal erosion. Nature-based solutions have been recognised as an opportunity to simultaneously address the coastal hazard risks and achieve biodiversity goals. While such solutions are included in climate adaptation strategies, “hard” engineered solutions are still often preferred by those implementing the schemes. We sought to explore the diverse perspectives on UK coastal flood risk management among interested and/or affected groups by utilising the Q-methodology. We identified five perspectives: (1) The Pro-Green Practitioners; (2) The Future-Planning Relocators; (3) The Case-by-Case Thinkers; (4) The Cautious Practitioners and (5) The Climate Change Concerned. All five perspectives strongly valued the co-benefits of nature-based solutions and their role in coastal risk reduction. None of the perspectives prioritised hard-engineered solutions as the primary flood protection strategy in the UK, though they recognised their role in protecting essential infrastructure. The main disagreements between perspectives were (1) on the need for relocation strategies, and (2) whether nature-based solutions could cause social inequalities. The Q-methodology does not identify how prevalent such perspectives are, thus further research is needed to assess the social acceptance of nature-based solutions.

## Graphical abstract

**Figure:**
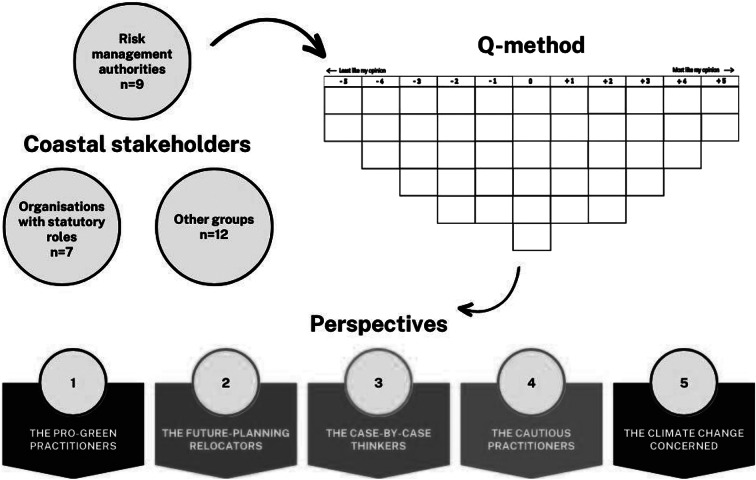

## Impact statement

Dynamic coastal zones are under pressure from sea level rise putting coastal communities at increased risk of flooding and coastal erosion. Historically, coastal protection in the UK has prioritised “hard” engineering such as seawalls, but since the introduction of Shoreline Management Planning, other strategies such as managed realignment have been increasingly considered. A hard engineering strategy is not financially feasible everywhere and would not allow for adaptation to coastal change under future climate scenarios. Nature-based solutions have gained growing attention as they can mitigate and adapt to climate change impacts, enhance biodiversity and contribute to people’s well-being. However, the implementation of such solutions is still relatively slow – even where they have been identified as the most sustainable option. Here we found that interested and/or affected groups such as risk management authorities, coastal partnerships and homeowners, express support for the direction of the UK national flood and coastal erosion risk management policy. All participants were convinced of the benefits of nature-based solutions. Yet, this study also highlighted that the current governance, appraisal and funding mechanisms are not fully equipped to consider the co-benefits of nature-based solutions and their role in coastal flood risk management. The core implication of this study is that stakeholders hold different perspectives/framings which affects the decision-making process. Whatever the strategies identified as technically optimal, the variety of framings held by different stakeholder groups need to be engaged to generate social acceptance, by identifying points of contention and agreement.

## Introduction

Rising global mean sea levels and increasing frequency of coastal inundation events are likely to increase the risk of coastal hazards such as flooding and erosion (IPCC, 2019). For example, damages to property from coastal flooding in England are projected to treble by the 2080s based on high-end scenarios of climate change (Sayers et al., 2022). Globally, even keeping current protection standards, estimated annual damage is expected to be USD 84 billion by 2050 under the RCP4.5–SSP2 scenario (Tiggeloven et al., 2020). In response to these risks, OECD (2019), IPCC (2022) and other international and national organisations urge countries to adapt to rising sea levels.

In the UK, there is a system of “Coastal Change Adaptation Plans” and “Shoreline Management Plans” (SMPs) at a broad scale which set strategic policy (DEFRA, 2006; Ballinger and Dodds, 2020; Kirby et al., 2021; Sayers et al., 2022; Scottish Government, 2023). At a more local scale, this policy is translated into a coastal scheme. The options for schemes can be considered along a spectrum from grey (hard engineering) to green solutions (that include natural and nature-based solutions [NbS]) (Schoonees et al., 2019). “Do nothing” or relocation of assets or people at risk are other key policy options.^1^ Studies show that even Neolithic communities 7,000 years ago used man-made defences to protect their settlements from sea-level rise (Galili et al., 2019). Yet, the rapid coastal development starting in the 19th century accelerated the use of seawalls and groynes, making grey structures the most common form of defence from coastal erosion and flooding (Wu and Barrett, 2022). While such structures can be part of an effective adaptation and flood risk management strategy, such static structures lack adaptability and often cause the adjacent areas to flood and erode (e.g., Cooper et al., 2020; Nunn et al., 2021). In response to the climate crisis, NbS have been proposed as actions to restore, protect and sustainably manage nature and simultaneously address societal challenges (e.g., Cohen-Shacham et al., 2016).

NbS is an umbrella concept that includes ecosystem-based approaches, restoration and recreation of natural ecosystems as well as hybrid approaches (Sutton-Grier et al., 2015; Seddon et al., 2020a; Bridges et al., 2021). At the coast, hybrid approaches include beach nourishment and managed realignment that may involve some engineering capabilities. Coastal ecosystems are not only biodiversity hotspots, but they also provide flood and coastal erosion protection. For example, saltmarshes are natural buffer zones and can reduce the height of the waves by 20% in storm surge events (Möller et al., 2014). Mangrove forests provide flood protection by buffering waves and storms (Menendez et al., 2020). Furthermore, NbS can also provide 37% of the mitigation efforts required by 2030 to reach the Paris Agreement targets (UNEP and IUCN, 2021). However, more than 60% of global mangroves (Goldberg et al., 2020) and 25–50% (McOwen et al., 2017) of global saltmarsh have been estimated to be lost or damaged, based on datasets since the 1970s, due to continuing pressures of urbanisation, coastal development, land use change and coastal squeeze.

Despite, NbS providing a “triple win” for climate, people and biodiversity (JNCC, 2021), their implementation is relatively slow, in particular, for coastal flood protection. One well-acknowledged limitation is funding. The UN Environment Programme’s State of Finance for Nature report showed that NbS are receiving only a third of the investment needed by 2030 to meet the Paris Agreement targets (UNEP, 2022). The other, previously less discussed, is the social acceptance of NbS. Studies show that the social acceptance of NbS for flood protection depends on the perceived fairness of solutions (Nóblega-Carriquiry et al., 2022), trust in implementers (Anderson et al., 2021), knowledge and perceived and relative effectiveness of NbS for flood protection (Needham and Hanley, 2019; Bernello et al., 2022). Yet, most studies do not attempt to give the participants the choice to evaluate the benefits and limitations of both NbS and grey structures, and select their preferred option given the options across the grey–green spectrum. Therefore, this study seeks to understand what type of coastal flood protection schemes are preferred, why and by whom. By understanding the “framings” which different groups hold, there is an opportunity to engage those perspectives, debate the evidence, and overcome the challenges which are preventing the implementation of sustainable coastal policies.

## Methods

To elicit the perceptions and priorities of flood protection schemes, we used the Q methodology, also known as Q method or simply as Q. It is a mixed method based on both quantitative and qualitative research principles (Ramlo, 2023). It was developed by British physicist and psychologist William Stephenson (1902–1989) based on correlational and factor analyses (Brown, 1980). Q methodology aims to explore people’s perspectives and categorise them into clusters based on their values and mental models and thus is often described as a semi-quantitative study of subjectivity (Watts and Stenner, 2014; Zabala et al., 2018; Ramlo, 2023). Participants are asked what is important and meaningful from their personal perspective and the meanings of configurations are attributed *a posteriori* through interpretation (Coogan and Herrington, 2011). It does not require a large sample size as its aim is to identify the various viewpoints on the research subject rather than estimate their prevalence (Brown, 1980). Q methodology has been applied to explore stakeholder perceptions on various environmental management themes such as wildland management (Deary and Warren, 2018), dam planning (Schulz and Adams, 2020), habitat restoration (Zenone et al., 2021), ecotourism (Lee, 2022), flood management (Tafel et al., 2021) and scenario planning (Jiren et al., 2023). It is also a useful method for exploring controversial topics as it is less confrontational than surveys or interviews, but still allows eliciting differing opinions (Churruca et al., 2021). In the Q-methodology procedure, participants are asked to sort and rank a set of statements that has been obtained from a relevant large data set. There are five main steps to the Q methodology that are described in detail in the following sections: (1) Obtaining a range of perspectives on the research topic (concourse); (2) Developing the final set of statements (Q-set); (3) Selecting the participants (P-set); (4) Sorting the statements (Q-sort) and (5) Statistical analysis and interpretation of factors.

### Concourse and Q-set

The concourse is an extensive set of statements that reflect a range of perspectives on a given issue, which in this study was obtained a priori through a literature review of academic papers, strategies, reports, and blog posts (*n* = 24). The initial concourse consisting of 364 statements was then organised into eight emergent themes: benefits of NbS, benefits of grey defences, benefits of managed realignment, benefits of hybrid solutions, limitations of NbS, limitations of grey defences, limitations of managed realignment, limitations of hybrid solutions, short term political decisions, stakeholder engagement. After removing duplicates and statements that were not directly relevant to the research objectives, a final set of 44 statements, the Q-set, was developed (Supplementary Table S1), with most of the statements extracted verbatim but standardised for syntax. To test the clarity and comprehensiveness of the statements, eight project researchers participated in a pilot in-person sorting process (Q-sort procedure explained in section “Procedure”, excluding pre-sort survey and post-sort interview). Individual statements were then edited or replaced, based on the informal feedback received, to ensure a comprehensible and comprehensive set of statements on various coastal management schemes.

### Participants

Participants (called the P-set) for this study were selected purposively (Watts and Stenner, 2012) based on a typology of roles identified for flood and coastal erosion risk management (Environment Agency, 2020) – risk management authorities (e.g., government agencies and local authorities), organisations and people with statutory roles (e.g., coastal partnerships, regulators, agencies and land owners) and other organisations and people affected by coastal hazards (e.g., residents, developers, insurers, infrastructure providers, businesses). We also included academia and environmental consultancies due to their influence on decision-making. We expected that these interested and/or affected groups would have different priorities and perspectives. Study participants were recruited via emails. In total, 87 invitational emails were sent between December 2022 and February 2023 to representatives of the above-mentioned groups in England, Wales and Scotland. Landowners and homeowners were recruited through newsletters and social media.

The final sample consisted of 31 participants (16 female, 15 male) representing various risk management authorities, non-governmental organisations (NGOs), coastal partnerships and communities from England (15 participants), Scotland (15 participants) and Wales (1 participant) (Table 1). Three organisations had a coastal heritage focus, and one organisation had a nature conservation role. Two NGOs could also be described as land managers. Four residents owned the property they lived in, while one resident was a long-term renter of a coastal property.

**Table 1.** Interested and/or affected groups represented in the P-set

Sector	No. of organisations	No. of individual participants
Risk management authorities		
Government agencies	5	6
Local authorities	4	4
Organisations with statutory roles		
Coastal partnerships	4	4
NGOs^a^	3	5
Other groups		
Government agency^b^	1	1
Environmental consultancies	2	2
Academia	4	4
Residents	5	5
Total	28	31

a Two NGOs are also involved in land management.

b This government agency has a different remit, therefore is not included under the risk management authorities.

### Procedure

**Figure 1.**
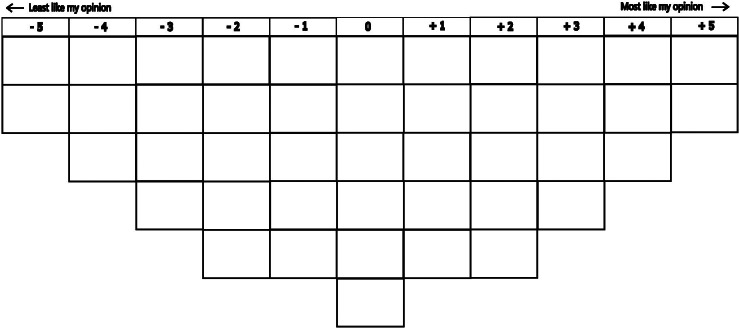
Q-grid in a shape of normal distribution designed for 44 statements. The statements are allocated in columns ranging from -5 (indicating relative disagreement) to +5 (indicating relative agreement). The vertical position within the column, i.e., row is not important and does not prescribe a level of agreement or disagreement

The sorting process, called the Q sort, was conducted online using the QMethodSoftware (Lutfallah and Buchanan, 2019). Before proceeding with the Q-sort, study participants were asked to complete a short demographic survey and pre-sort the statements into three categories – agree, neutral and disagree. Next, the participants were asked to place statements in a Q-sort structure (Q-grid) in columns ranging from +5 (most like my opinion) to −5 (least like my opinion) (Figure 1). The vertical position within the column, that is, row does not affect how the statement is weighted or analysed. These values are relative and negative values do not necessarily mean that the participant fully disagrees with those statements. The study adopted commonly used forced normal distribution, which means that participants were only allowed to place the statements in the pre-designed slots of a symmetrical grid. Non-standartised or free distribution, while not common, is possible (Watts and Stenner, 2012), but none of the participants insisted on such distribution. The participants were asked to reflect their own personal opinions rather than those of their affiliated organisations’ when sorting the statements. A post Q-sort interview was conducted with each respondent to understand their rationale for placing the statements in the grid (Gallagher and Porock, 2010).

### Data analysis

The obtained Q-sorts were analysed with the KADE software v1.2.1 (Ken-Q Analysis Desktop Edition) (Banasick, 2019). Firstly, a correlation matrix was produced to identify similarities and differences between participants’ Q sorts. Secondly, as mentioned earlier, the goal of Q methodology is to categorise peoples’ perspectives into clusters based on their values and a factor analysis was used to achieve this. We chose centroid factor analysis based on the correlation matrix as it is the oldest factor extraction technique allowing for more data exploration compared to the principal component analysis (Watts and Stenner, 2012). Initially, eight factors were identified by default. Of those, five perspectives had eigenvalues greater than 1.00 (Watts and Stenner, 2012) and thus a five-factor solution was selected. Thirdly, a Varimax rotation was applied to the selected perspectives producing a table with factor loadings by participants. Varimax rotation is a way of simplifying the complexity of the data to draw out its structure by minimising the number of variables with high loadings (Akhtar-Danesh, 2017). Factor loadings indicate the association between the participant and each identified factor. Finally, the software produces a set of tables with the *Z*-score ranking for each statement and reconfigured Q sorts (factor arrays) are built for each perspective (factor) based on the composite and weighted *Z*-scores from all the participants who define a particular factor (Zenone et al., 2021). “Distinguishing statements” for each perspective are identified along with the consensus statements that are ranked similarly by all participants. Post Q-sort interviews were transcribed and analysed in NVivo 1.5.1 to identify individual rationales and enable interpretation of why participants held certain framings. A flexible coding approach was used to qualitatively analyse the interview transcripts (Deterding and Waters, 2021). Firstly, they were analysed deductively by coding the answers regarding each statement. Secondly, transcripts were analysed again inductively for other themes which inferred people’s reasons for holding their perspective. The interpretation of factors is based on the individual and factor array configuration of statements, distinguishing and consensus statements, and qualitative data obtained during the post-sort interviews. The researcher plays an active role in the Q methodology as the interpretation relies on the researcher’s knowledge and experience of the study topic (Zabala et al., 2018).

## Results

**Figure 2.**
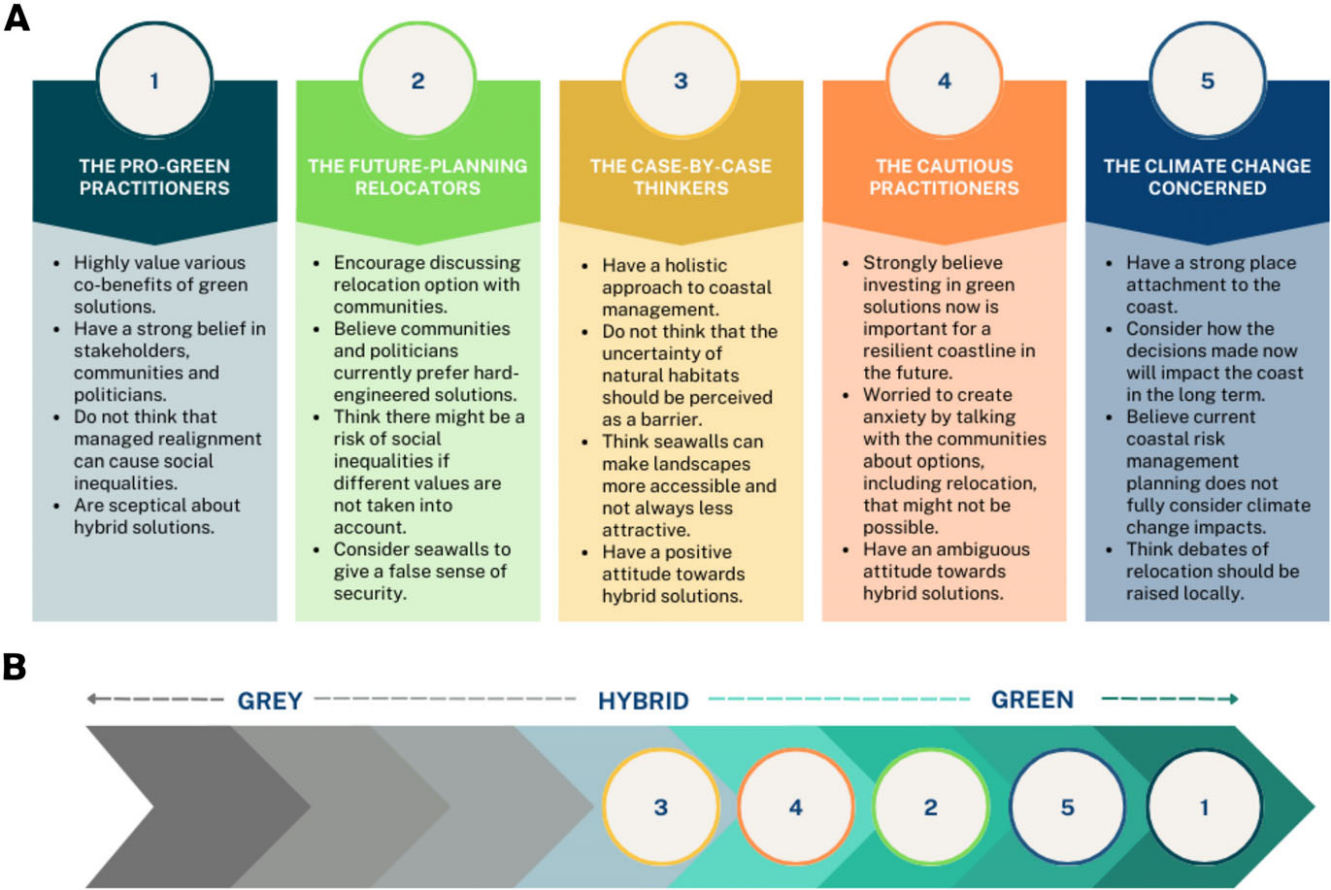
Five perspectives identified in this study. A: description of each perspective. B: Placement of each perspective according to their strength of agreement to statements representing solutions on a “grey to hybrid to green” scale.

Based on the configuration of statements, factor arrays and post-sort interviews, five distinctive perspectives were identified: (1) The Pro-Green Practitioners; (2) The Future-Planning Relocators; (3) The Case-by-Case Thinkers; (4) The Cautious Practitioners and (5) The Climate Change Concerned (Figure 2). The labels refer to the most distinguishing characteristics of each perspective. The five-factor solution explained 46% of the total variance^2^ (Supplementary Table S2). Of the total 31 Q-sorts, 11 did not load significantly (*p* > 0.05) on any of the perspectives (Supplementary Table S3), thus were not included in the factor interpretation, but are considered in general discussion. Table 2 illustrates idealised Q-sort values for each perspective and identifies distinguishing and consensus statements. When reading the results text below, cross-reference to Table 2 to identify the question numbers, and see further detail in the Supplementary Material.

**Table 2.** Idealised Q-sort values for five perspectives

No.	Statement	P1	P2	P3	P4	P5	Distinguishing or consensus
1	Natural habitats such as sand dunes, beaches, salt marsh, and cliffs provide a natural barrier to flooding erosion around the coast	4	1	5	2	4	
2	Nature-based solutions offer a wide assortment of social benefits and community interests such as recreation and well-being	5	−1	1	3	2	Distinguishing for F1 and F2
3	Natural habitats can adapt to changes in climate and self-repair after major storm events	3	0	2	2	4	
4	Nature-based solutions may provide carbon storage and biodiversity improvements	5	0	2	5	3	Distinguishing for F1, F2 and F4
5	Taking proactive steps to create nature-based solutions now will save money in the future and help to create a coastline that is naturally resilient to future changes	1	3	4	4	5	Distinguishing for F1
6	Public opinion currently supports installing, maintaining and funding hard-engineered flood protection structures	0	2	−2	−5	−3	Distinguishing for F1 and F2
7	Hard-engineered adaptation has reduced the human death toll from disasters	−1	−4	0	1	−4	
8	If designed appropriately, seawalls can increase local ecosystems and biodiversity’s resiliency	−4	−3	0	4*	0	Distinguishing for F4
9	A seawall provides a high degree of protection against coastal flooding and erosion	−3	−5	−1	−2	−4	
10	The high level of security provided by a seawall can favour the development of the areas further inland	−1	−2	−2	0	−5*	Distinguishing for F5
11	Managed realignment can help avoiding scenarios which necessitate long-term financial commitment	0	−4	3*	0	−3	Distinguishing for F3
12	Managed realignment can create a new habitat area that acts as a natural buffer to coastal waves and is much cheaper to maintain over the long term	1	−2	4	0	3	
13	Managed realignment could be the only viable option in the long term for some coastal areas	3	3	3	−3*	2	Distinguishing for F4
14	Managed realignment can enable more outdoor activities in nature, which could contribute to resident well-being	2	−1	1	1	2	Distinguishing for F2
15	Managed realignment has the potential to deliver multiple co-benefits	4	2	5	3	0*	Distinguishing for F5
16	Managed realignment builds resilience and reduces the impact of coastal hazards on infrastructure	0	−2	3	−1	3	
17	The co-benefits of restoring natural environments are hard to quantify	−1	−4	0	−1	−5	
18	In the case of restored ecosystems, it can take a long time for ecosystems to get established for the natural systems to provide the necessary level of coastal protection	−4	0	−4	2	−2	Distinguishing for F4
19	Permitting for natural projects can be a more difficult process than for built projects	−3	0	2	4	−2	Distinguishing for F3 and F4
20	There is a lack of complete information about the costs and effectiveness of projects that restore or manage habitats for coastal protection	−2	−2	−2	−2	−1	Consensus
21	Natural habitats are dynamic and introduce uncertainty that could be a barrier to the wider use of natural habitats in coastal defence planning	1	1	−4	1	−3	Distinguishing for F5
22	The more nature-based a solution is, the higher its demand for land	−5	−3	−5	−4	−4	
23	Protecting all coastal locations through hard defences where currently planned is not likely to be cost-effective, nor financially realistic	2	4	−1	5	1	Distinguishing for 3
24	Hard-engineered structures can lull communities into thinking they are safe from all disasters leading to increased loss of life or property	−2	4	1	0	2	Distinguishing for F1
25	Hard structures simply deflect wave energy to adjacent areas	−4	−3	−5	−3	3*	Distinguishing for F5
26	Grey structures are continuing to be built with little positive improvement in practises or management	2*	−1	−4	−3	−2	Distinguishing for F1
27	Seawalls can reduce the attractiveness of the landscape	−1	1	−3	−1	4	Distinguishing for F3 and F5
28	Seawalls can destroy natural habitats such as intertidal beaches and dune systems	1	3*	−2	0	1	Distinguishing for F2 and F3
29	Managed realignment requires significant up-front costs and long-term planning and community engagement	1	0	−1	−4*	0	Distinguishing for F4
30	The process of implementing managed realignment policy can result in social inequalities	−5	2*	−2	−5	−2	Distinguishing for F2
31	Communities are likely to be surprised and angered by coastal adaptation policies that do not “hold the line” on existing defences	−3	3*	0	−4	−1	Distinguishing for F2 and F4
32	Managed realignment can result in the loss of land area which provides livelihoods to farmers	−2	−5	−3	−2	2*	Distinguishes F2 and F5
33	A combination of green and grey infrastructures can significantly reduce flooding	2	−2	0	2	−1	
34	Hybrid solutions can be used in areas where there is little space to implement natural approaches alone	0	−1	−1	1	−1	Consensus
35	Within urban environments, hybrid solutions can support resilience to climate change	0	−1	3*	−1	0	Distinguishing for F3
36	Modified seawalls and other hybrid structures are often resource and energy-intensive and lack the capacity to adapt to sea level rise	−1	1*	−3	−3	−3	Distinguishing for F2
37	Hybrid systems, due to the built part of them, can still have some negative impacts on species diversity	2*	−3	−3	−1	0	Distinguishing for F1
38	Politicians prefer short-term results compared to long-term ones due to the political cycle and the effort to be re-elected	−2	5	2	1	0	Distinguishing for F1
39	Long-term strategies are needed to facilitate cost-effective and rapid implementation of integrated flood management	4	4	1	3	1	
40	Most adaptation is reactive rather than proactive with the lack of consideration of climate change impacts in coastal planning	−3	0	−1	−2	5*	Distinguishing for F5
41	Risk management authorities can help ensure that the natural environment contributes to improving flood and coastal resilience by working closely with those creating/restoring natural habitats	3	2	1	2	1	Consensus
42	Stakeholders often tend to fall back on affordable and familiar practices which are less risky and more predictable in their outcomes	−2	1	0	3	−1	
43	Coastal communities need to be engaged to plan for their future over several decades, but the capacity and political will to do so does not currently exist	0	2	4	0	1	
44	Public debate should be increased on the possibility and potential need for future relocation of properties and communities	3	5	2	−2	−2	

*Note:* Distinguishing statements (*p* < 0.05: Asterisks indicate significance at *p* < 0.01).

The results of the demographic survey showed that nearly half (48%) lived a 0–2 km distance from the coast, while 19% lived more than 20 km away from the coast. 90% of participants used the coast for activities such as coastal path walking, wild swimming or were involved in environmental activities (e.g., beach cleaning, restoration projects). Two representatives of local communities had higher education (secondary school/A-levels), but the rest of the participants had either undergraduate degrees (35.5%) or postgraduate degrees (58.1%).

### Perspective 1: The pro-green practitioners

Seven participants from two government agencies, two NGOs, both consultancies and one coastal partnership were significantly associated with this perspective (Supplementary Table S3). This is the most dominant perspective, with an eigenvalue of 8.7 and explaining 14% of the total variance. The participants associated with this perspective can be described to have a pragmatic approach with a preference for NbS. This perspective highly valued the co-benefits that NbS, including managed realignment (Question number 15: Idealised Q-sort value for the factor: +4), can provide, such as enhanced biodiversity, carbon sequestration (4: +5) and social benefits for well-being and recreation (2: +5; 14: +2) (Supplementary Table S4). Participant 29 talked about how NbS *“…[are] better for communities’ well-being as opposed to having just concrete*”. This perspective was the only perspective that overall believed that grey structures are too predominant in the solutions being offered, with little improvement in practice (26: +2) although one recognised that guidance has improved with investment in low-carbon technology and consideration of sustainability for coastal infrastructure. This perspective was the only one to hold an opinion that hybrid systems can have negative impacts on species diversity (37: +2) by continuing to rely on seawalls (Participant 17).

This perspective has a relatively strong trust in stakeholders (for instance, other risk management authorities), communities and politicians (24: −2, 38: −2, 42–2), and the decisions they make compared to other perspectives. Participant 29 expressed that statement 24 underestimates how much coastal communities are aware of flood risks and their knowledge about the level of protection hard defences provide. Perspective 1 strongly disagreed that managed realignment could result in social inequalities (30: −5) and that NbS are land intensive (22: −5). They also did not think that ecosystems should take a long time to re-establish (18: −4), sharing this view with Perspective 3. This perspective was neutral regarding the public support for hard-engineering solutions (6: 0). Participants 16 and 29 mentioned that it depends on the section of the public, but that in general, people are much more supportive of natural solutions.

### Perspective 2: The future-planning relocators

This perspective was held by 3 participants from academia, local authority and government agency which was not a risk management authority. This perspective has an eigenvalue of 1.7071 and explains 8% of the study variance. This perspective strongly believed that there should be increased debates about relocation (44: +5) (Supplementary Table S5). Participants of this perspective considered the decision-making process and political debates more than any other perspective. This perspective believed that politicians prefer short-term results (38: +5). Referring to statement 44, participant 7 said that “*politicians are avoiding those questions, avoiding the uncomfortable truths*”. In contrast to Perspective 1, this perspective agreed that hard-engineered structures can lull communities into a false safety (24: +4). More than any other perspective, perspective 2 thought that the public supports hard engineering (6: +2) and the communities will be dissatisfied if the coast is no longer defended (31: +3). Furthermore, they also believed that the process of implementing managed realignment could result in social inequalities (30: +2). Participant 18 commented that while it might not always happen, there is a significant risk if communities are not engaged enough and if different values of the landscape are not taken into account.

This perspective gave less attention to the statements about the co-benefits of NbS (2: −1; 4: 0) and the potential of managed realignment to create a habitat area that acts as a buffer (12: −3), enable outdoor activities (14: −1) and build resilience (16: −1). This does not, however, mean that they disagree with these statements fully, but political and decision-making aspects were more important points of discussion for this perspective. This perspective strongly disagrees that managed realignment could result in a loss of land area providing livelihoods (32: −5), mentioning the opportunities managed realignment can offer to farmers such as a more controlled flooding allowing grazing to take place. In addition, Participant 7 expressed that seawalls only give limited protection (9: −5) for a limited time: “*I just think it can lead to a false sense of security … rather than having to adapt to changing climate*”.

### Perspective 3: The case-by-case thinkers

The four participants that significantly loaded onto this perspective, represent academia, residents, coastal partnerships and local authorities. This perspective explains 11% of the total variance with an eigenvalue of 1.4279. This perspective is characterised by having a more holistic view of coastal management and applying a case-by-case approach. The role of natural habitats in flood protection was important to this perspective (1: +5) (Supplementary Table S6), stating that it is “*fairly self-evident*” that natural habitats can regenerate without human intervention after storm events (Participant 26). Perspective 3 had a positive attitude towards managed realignment and its role in flood management in the future (11: +3; 12: +4; 13: +3,15: +5, 16: +3). Besides, this perspective was less sceptical about hybrid solutions (35: +3) compared to other perspectives, disagreeing with statements 36 (−3) and 37 (−3) that suggest that modified seawalls and hybrid systems lack the capacity to adapt and have negative impacts on biodiversity. However, Participant 21 acknowledged that there will be some trade-off with biodiversity, though not necessarily *“…as big a trade off as that statement makes it look*”.

This perspective disagreed that the dynamism of natural habitats introduces uncertainty that could be a barrier for flood defence (21: −4). Participants 9 and 26 argued that uncertainty is inherent, including for hard-engineered structures, so this should not be insurmountable. Participant 21 added that the challenge is the political setting and conversations with communities. Perspective 3 was the only perspective which disagreed that seawalls can reduce the attractiveness of the landscape (27: −3), mentioning seafronts in Dundee and London, where they have created an accessible urban frontage that connects a city to the sea and pointing out that managed realignment schemes would not be suitable for these urban locations.

### Perspective 4: The cautious practitioners

This perspective was held by 3 participants of which two represented local authorities (Scotland and England), and one represented a coastal partnership in England. This perspective has an eigenvalue of 1.2307 and explains 5% of the total variance. This perspective, similarly, to perspective 1, has a pragmatic view of flooding and coastal erosion schemes, but is more cautious regarding public engagement and what schemes are possible to implement. They strongly believed that protecting all coastal locations through hard defences will not be possible (23: +5) and that taking proactive steps now to invest in NbS, will save money and create a more resilient coastline in the future (5: +4) (Supplementary Table S7). Participant 8 commented saying that “*we need to make space now for our coastal resilience needs in the future*” not only through flood and coastal erosion risk management planning but also land use planning. Perspective 4 considered the process of implementing NbS more than any other perspective, characterising current legislation as prohibitive and working against nature instead of protecting it (19: +4). This perspective was the only one that slightly agreed that ecosystem restoration might take a long time (18: +2). However, while Participant 14 agreed quite strongly saying they tend to take a long time, with some cases established relatively quickly, Participant 8, on the contrary, said, “*It’s amazing how fast they do actually get populated and start producing biodiversity benefits and other benefits*”.

This perspective has an ambiguous view towards hybrid solutions. Participants who loaded on to this perspective slightly agreed that a combination of green and grey can benefit flood protection (33: +2) and can be used in smaller spaces (34: +1) but disagreed that they can support resilience in urban areas (35: −1). This perspective did not think that managed realignment requires significant up-front costs and community engagement (29: −4), at the same time being sceptical about engaging with the communities (43: 0) or raising public debates about relocation (44: −2). Participant 14 commented that this might increase anxiety and the levels of expectation in case there is no funding available for certain projects, at the same time stating that local communities recognise that there might be a need for different solutions in places where current protection schemes no longer provide protection.

### Perspective 5: The climate change concerned

This perspective comprises three participants, all from Scotland – two local residents and a Scottish-based NGO. This perspective explains 8% of the study variance with an eigenvalue of 1.2328. This perspective is characterised by having a more emotional connection to the coast. They look at the bigger picture, how the decisions we make at the coast can impact nature in the longer term. These participants are very concerned about climate change. They strongly believed in taking proactive steps (5: +5) and agreed that currently, most adaptation is more reactive rather than proactive and that coastal planning does not sufficiently consider climate change (40: +5) (Supplementary Table S8). Participant 23 says that there is no long-term planning where they live and that local authority tends to make decisions that are based on the current conditions rather than considering climate change projections. Thus, it was important for this perspective that NbS can adapt and self-repair (3: +4) and that natural habitats provide a barrier to flooding and erosion (1: +4). In contrast to Perspective 3, they strongly believed that seawalls reduce the attractiveness of the landscape (27: +4) and that hard structures simply deflect wave energy (25: +3). Participant 30, however, acknowledged that sometimes a seawall could be the only option to defend a heritage site of high historical and social value, or important infrastructure.

Perspective 5 doubted that hard engineering has reduced the human death toll (7: −4). Participant 22 commented that the current risk of fatalities from coastal flooding does not seem to be a major problem in the UK and that it is important to “[look] *after nature in order to look after the future of mankind anyway.* *We’ve been too selfish for too long*”. Regarding potential relocation, the participants of this perspective considered their location (44: −2). Participant 30 commented that in Scotland “*it would be counterproductive to raise the public debate to that sort of level because the actual number of people it will affect is so small that you could create a sort of panic which is not really justifiable*” yet agreed that it is necessary for other locations such as parts of England. The participants of this perspective did not think that co-benefits of restoring natural environments are hard to quantify (17: −5), although participant 23 said it might be true yet that the “*positives may not appear immediately*” due to the dynamic nature of the coast.

### Consensus statements

There are three statements (20, 34, 41) which most participants ranked similarly. All participants mildly disagreed that there would be a lack of complete information about the costs and effectiveness of habitat restoration projects (20). Two participants (Participants 18 and 21) said that such argument might be used to justify not implementing NbS. Participant 21 acknowledged the situation seems to be improving. Statement 34 on the use of hybrid solutions was ranked between mild disagreement (−1), ambivalence (0) and mild agreement (+1). Generally, participants gave low ranking to the statements they did not know enough or were not sure about. These statements also received less attention in post-Q-sort interviews. Statement 41 on risk management authorities received mild agreement from all participants. Participant 29 highlighted that “*[…] authorities if they’re engaging with other groups who have specific knowledge in the types of habitats or environment of a certain area, then that should, improve the decision making and therefore the types of engineering that are used in that area, and then hopefully then improve the flood and resilience of that coastline*”. Participant 21 highlighted that risk management authorities already work very closely with each other. Although statement 22 stating that NbS can be land demanding, was not identified as statistically significant consensus statement, all perspectives disagreed with it.

### Reflections on the method

The Q-methodology relies on a small purposive sample size and the results cannot be extrapolated to a wider population (Brown, 1980; Webler et al., 2009; Watts and Stenner, 2012). Furthermore, the ranking is relative and depends on the overall Q-sort and might not reflect true values (Webler et al., 2009). Therefore, post-sort interviews can give more insight into the ranking strategy and prioritisation and inform factor interpretation to minimise researcher bias (Gallagher and Porock, 2010). We highly recommend this combination of mixed methods. In general, participants gave positive feedback on this approach during the post-sort interview, stating that the ranking exercise gives a structure and compels reflection on the relative importance of issues. However, participants commented that some of the statements were too general and would require qualification or more contextual information to make a final decision in context.

## Discussion

We identified five perspectives on coastal flood risk management among interested and/or affected groups. Based on the overall statement ranking, all perspectives prefer NbS and their provided co-benefits (Figure 2B). There is ambiguous acceptance of hybrid solutions and while none of the perspectives favour fully defending the coast with hard-engineered structures, there are varying opinions about seawalls among the participants. Below we discuss the main salient and consensus themes identified by participants in post-sort interviews.

### Opportunities and limitations of coastal nature-based solutions

The appeal of NbS can be attributed to their ability to provide various co-benefits by not only solving environmental issues but also addressing social challenges and enhancing biodiversity (Cohen-Shacham et al., 2016; Gómez Martín et al., 2020; Seddon et al., 2020a, 2020b; Jordan and Fröhle, 2022). All participants were aware of the co-benefits of NbS and highly valued them as has been described in the literature (Tafel et al., 2021; Riegel et al., 2023). Yet, other studies have reported decision makers to possess limited knowledge on the co-benefits of NbS thus hindering their implementation for flood management (Wells et al., 2019; Solheim et al., 2021). The contributions of NbS to social well-being can sometimes be limited by restricted public access, or appeal only to a specific set of users such as birdwatchers. Including all co-benefits and potential disbenefits enables transparent decision-making process (Ruangpan et al., 2021; Curt et al., 2022).

One of the barriers to implementing NbS effectively in policy and practice often is the limited information on the cost-effectiveness of NbS and challenges in measuring co-benefits (Ürge-Vorsatz et al., 2014; Seddon et al., 2020a). Marine and coastal systems and the ecosystem services they deliver are particularly complex and dynamic and often less understood than their terrestrial counterparts (O’Leary et al., 2023). There was a consensus in this study that at least in the UK that the evidence base is improving whilst not always being easily accessible for everyone. Ommer et al. (2022) have summarised approaches that quantify indicators of air and water quality, habitat quality and biodiversity as well as indicators for job opportunities, tax revenue and social inclusion/exclusion. Despite the availability of such tools, there is still limited evidence on non-tangible non-market human and nature benefits such as health and well-being (Dick et al., 2020; Viti et al., 2022).

One of the most debated statements in this study was “the more nature-based solution is, the higher its demand for land” (Hartmann et al., 2019). Hartmann et al. (2019) state that private land is critical for implementing NbS for coastal flood risk management and NbS are generally more land intensive than grey solutions. This was strongly contested by the respondents mentioning that such a generalisation ignores the variety of NbS. It was acknowledged that certain schemes such as managed realignment can be more land intensive, but that this can often be justified where benefits overweigh the loss. Participants highlighted opportunities presented for alternative livelihoods such as salt marsh grazing and tourism (McKinley et al., 2020). However, none of the participants identified themselves as farmers. Managed realignment schemes are generally supported by residents and local organisations, yet not preferred by farmers due to potentially suffering from economic losses and desire to maintain their agricultural heritage and identity (Liski et al., 2019). Furthermore, in the UK the land for managed realignment is normally bought off from the landowners and up to date, there are no reported cases of compulsory purchase of land. Land acquisition costs are always considered in project designs and often are a significant proportion of costs (Hudson et al., 2015). That was also the reason most perspectives disagreed that managed realignment could result in social inequalities. However, NbS are not inherently just and socially inclusive (Haase, 2017), therefore while the implementation of such solutions itself might not cause social inequality, it could exacerbate the existing injustices. Other literature highlights a problematic potential for NbS to be used as “greenwashing” where a priority on emission offsetting and exclusion of communities from decision processes leads to sub-optimal outcomes (e.g., Pascual et al., 2014; Almanza-Alcalde et al., 2021; Seddon, 2022). Thus, integrating justice principles and promoting communication, collaboration and stewardship is essential (Anguelovski and Corbera, 2023; O’Leary et al., 2023).

### The role of grey and hybrid structures in coastal flood risk management

Across all five perspectives, we found an unambiguous agreement that the UK coast cannot and should not be protected through hard engineering solutions alone. This, however, does not imply that seawalls and other solutions are not required at all. Yet, participants highlighted the still often prevalent and problematic perception that seawalls can defend or provide complete protection against flooding, while they only reduce the risk of flooding rather than eliminating it completely. The majority of flood defences in the UK provide a 1 in 100 or 1 in 200 year standard of protection, while in the Netherlands it varies between 1:300 and 1:100,00 years (Bisaro et al., 2020). This belief can provide a false sense of security and also make communities question the effectiveness of NbS for coastal flood risk management. The high number of casualties of the 2011 Great East Japan Earthquake and the Tōhoku Tsunami in Japan has partially been attributed to the false sense of security of seawalls (Strusińska-Correia, 2017; Boret and Gerster, 2021). Seawalls encouraged development in vulnerable areas, while the damage and death rate were high for areas with seawalls less than 5 m in height (Nateghi et al., 2016). For Small Island Developing States seawalls can be maladaptive if implemented uncritically based on other countries’ examples and not maintained regularly (Nunn et al., 2021). Although these examples do not necessarily reflect the UK hydrological, seismological, climatological and economic conditions, they help highlight the disastrous consequences of such discourse. Technological advances in design and materials together with advanced numerical models have improved contemporary seawalls (Williams et al., 2016). However, as all perspectives emphasise – seawalls are only effective if they are regularly maintained and repaired.

Hybrid solutions such as living walls or combining hard engineering with NbS, received equivocal perceptions about their role in flood risk management and climate change adaptation. One of the points of disagreement was that “greening the grey” approach does not contribute significantly to climate adaptation and only improves biodiversity at a small scale. Ecological enhancements (textured tiles or rock positioning) of hard coastal defences have been shown to support more diverse assemblages (e.g., Coombes et al., 2015; MacArthur et al., 2020; Kosová et al., 2023; Naylor et al., 2023). Yet, the patch-scale of enhanced marine artificial structures has been found not to have a universally positive effect on biodiversity (Strain et al., 2020). Furthermore, hybrid solutions that use features to enhance biodiversity still very much rely on the built part for flood protection (Rubinato et al., 2020). Hybrid solutions that are a mix of grey and green approaches in one location are reported to be more impactful (Palinkas et al., 2022) but still can have a negative impact due to the hard engineering (Sutton-Grier et al., 2015). Overall if the existing infrastructure does not allow for NbS, a mix of options or integrated greening of grey infrastructure even applied retrospectively can be an important strategy. Hybrid approaches, such as integrating vegetation in front of levees and seawalls can significantly reduce the costs and provide efficient flood protection (Du et al., 2020; van Zelst et al., 2021). Yet researchers warn and our participants concur that it should not be used as a Trojan horse to justify new development (Firth et al., 2020).

### Planned relocation: The most contested option for climate change adaptation at the coast

The final disagreement between the perspectives was whether a relocation of coastal communities will be required and to what extent. Planned relocation is a form of mobility in response to climate change first recognised by the Cancun Adaptation Framework agreement of COP 16 (UNFCCC, 2011). Compared to migration and displacement, planned relocation is small-scale and usually takes place within national borders. Fiji is at the forefront in this debate globally with a plan to relocate ~800 villages due to sea-level rise (GIZ, 2019). An early example of planned relocation was Vunidologoa, a village of 140 people relocated in 2014 (McNamara and Des Combes, 2015). To effectively implement such plans, it is important to have longitudinal studies and understand the impacts of relocation on livelihoods (Piggott-McKellar and Vella, 2023) and health (McMichael and Powell, 2021), beyond just financial considerations. In Simbach, Germany, 11 households were relocated after a millennial flood event that claimed five fatalities to use the land for flood protection measures (Mayr et al., 2020). This relocation was successful due to extensive personal communication and compensation. Meanwhile, in Austria relocation strategies have not been similarly successful mainly due to insufficient engagement with citizens, lack of knowledge transfer and limited flexibility of compensation (Thaler et al., 2020).

Perspective 2: The Future Planning Relocators felt strongly that relocation debates should be set on a national level and such a scenario is unavoidable. Meanwhile, Perspective 5: The Climate Change Concerned with all participants from Scotland thought that such discussions should be regional where relocation might be required, for example, on predominantly low-lying, soft sedimentary coasts. In Scotland, relocation of at-risk assets (though not communities) has been considered (Garft et al., 2023). Sir James Bevan, the chief executive of Environment Agency, in his speech at Flood & Coast Conference 2022, urged admission of the inconvenient truth that due to climate change and sea level rise some communities will have to relocate (Bevan, 2022). In the UK context, relocation is not an SMP policy option, yet the policy option “no active intervention” in the Plans means that there will be no further investment in coastal defence schemes (DEFRA, 2006) and thus relocation might be necessary. Fairbourne village in Wales was assigned such policy option and the village was planned to be decommissioned by 2054 by the local authority (Buser, 2020). However, the residents did not accept this decision and have been taking action to identify other potential solutions and refusing to leave (Gerretsen, 2022; Arnall and Hilson, 2023) and thus this decision is under reconsideration. Despite relocation being acknowledged by risk management authorities as a meaningful option for low-lying communities to adapt to >1 m projected sea level rise, the issue is proving politically intransigent.

### Implications and further research

Our results revealed an increased consideration of NbS for coastal flood risk management in the UK where feasible, acknowledging their multiple co-benefits and the inherent flood risk reduction benefits of natural habitats among all five perspectives. All perspectives recognised that the historical predominance of hard-engineered solutions is not a suitable flood protection strategy in the UK under future climate projections, but they play an important role in protecting significant settlements, infrastructure or heritage. Relocation, while acknowledged by risk management authorities and debated in the adaptation literature, is still relatively unimplemented strategy on a wider political stage and requires attention. All five perspectives and all interested and/or affected groups theoretically confirm the direction of national policy for flood and coastal erosion risk management (although the Q-methodology does not allow for generalisation to a population). However, despite having flood and coastal erosion risk management strategies and SMPs, participants argued that in practice, there is a lack of forward-thinking and long-term planning. The post-sort interview results highlighted that current governance, appraisal and funding mechanisms are not fully equipped to consider the co-benefits of NbS and their role in coastal flood risk management, and therefore require improvement and reform.

Globally, sustainable solutions are likely to be context-dependent due to the diversity of coasts. Despite this context dependency, to justify the sustainability of coastal solutions, international practitioners will need to consider trade-offs between: the reduction of risks from coastal hazards, biodiversity conservation, climate adaptation, climate mitigation and social benefits, as revealed in the five perspectives identified in this study. Further research is required to capture the opinions of different stakeholders such as farmers, industry and residents in higher-risk areas. The Q methodology is a powerful tool to elicit priorities and perspectives on a local and national scale and can be used for scenario planning in combination with other research methods.

## Supporting information

Apine and Stojanovic supplementary material Apine and Stojanovic supplementary material

## Data Availability

The authors confirm that the data supporting the findings of this study are available within the article and/or its Supplementary Material.

## Footnotes

1 Monitoring, flood warning, flood insurance, building/household level flood proofing and community level resilience strategies for disaster preparedness, can enhance resilience or manage flood risk, but do not constitute an in situ intervention with a coastal scheme.

2 The explained variance of the Q-methodology factor analysis applied to environmental management studies varies from around 40% (e.g., Huge et al., 2016; Schulz and Adams, 2020) to around 50–63% (e.g., Nóblega-Carriquiry et al., 2022; Venneman et al., 2022; Bavin et al., 2023).

## Supplementary material

The supplementary material for this article can be found at https://doi.org/10.1017/cft.2024.4.
