# Supplementary material for: Is the coastal future green, grey or hybrid? Diverse perspectives on coastal flood risk management and adaptation in the UK
Source: Camb Prism Coast Futur. 2024 Feb 12;2:e4. doi: 10.1017/cft.2024.4 (PMC12337589; doi:10.1017/cft.2024.4)
Supplement: Apine and Stojanovic supplementary material [file S2754720524000040sup001.docx]

Is the coastal future green, grey or hybrid? Diverse perspectives on coastal flood risk management and adaptation in the UK - Supplementary material

Table S1. The set of 44 statements used in this study. BNbS=benefits of nature-based solutions, BGD= benefits of grey defences, BMR=benefits of managed realignment, BHS=benefits of hybrid solutions, LNbS=limitations of nature-based solutions, LGD= limitations of grey defences, LMR=limitations of managed realignment, LHR=limitations of hybrid solutions, STPD=short term political decisions, SE=stakeholder engagement.

|  | Statement | Theme | Source |
| --- | --- | --- | --- |
| 1 | Natural habitats such as sand dunes, beaches, salt marsh, and cliffs provide a natural barrier to flooding and erosion around the coast. | BNbS | Welsh Government, 2020 |
| 2 | Nature-based solutions offer a wide assortment of social benefits and community interests such as recreation and wellbeing. | BNbS | Doelle and Puthucherril 2021 |
| 3 | Natural habitats can adapt to changes in climate and self-repair after major storm events. | BNbS | Morris et al., 2018 |
| 4 | Nature-based solutions may provide carbon storage and biodiversity improvements | BNbS | Narayan et al., 2016; Sutton Grier et al., 2015; Rendon et al., 2022 |
| 5 | Taking proactive steps to create nature-based solutions now will save money in the future and help to create a coastline that is naturally resilient to future changes. | BNbS | Climate Change Committee, 2018 |
| 6 | Public opinion currently supports installing, maintaining and funding hard-engineered flood protection structures. | BGD | Rennie et al, 2021 |
| 7 | Hard-engineered adaptation has reduced the human death toll from disasters. | BGD | Doelle and Puthucherril 2021 |
| 8 | If designed appropriately, seawalls can increase local ecosystems and biodiversity’s resiliency. | BGD | Doelle and Puthucherril 2021 |
| 9 | A seawall provides a high degree of protection against coastal flooding and erosion | BGD | ClimateADAPT, 2016 |
| 10 | The high level of security provided by a seawall can favour the development of the areas further inland | BGD | ClimateADAPT, 2016 |
| 11 | Managed realignment can help avoiding scenarios which necessitate long term financial commitment | BMR | Climate Change Committee, 2018 |
| 12 | Managed realignment can create a new habitat area that acts as a natural buffer to coastal waves and is much cheaper to maintain over the long term. | BMR | Climate Change Committee, 2018 |
| 13 | Managed realignment could be the only viable option in the long term for some coastal areas. | BMR | Liski et al., 2019 |
| 14 | Managed realignment can enable more outdoor activities in nature, which could contribute to resident wellbeing. | BMR | Liski et al., 2019 |
| 15 | Managed realignment has the potential to deliver multiple co-benefits. | BMR | MacDonald et al., 2017 |
| 16 | Managed realignment builds resilience and reduces the impact of coastal hazards on infrastructure. | BMR | Williams et al., 2018 |
| 17 | The co-benefits of restoring natural environments are hard to quantify. | LNbS | Climate Change Committee, 2018 |
| 18 | In the case of restored ecosystems, it can take a long time for ecosystems to get established for the natural systems to provide the necessary level of coastal protection. | LNbS | Sutton Grier et al., 2015 |
| 19 | Permitting for natural projects can be a more difﬁcult process than for built projects. | LNbS | Sutton Grier et al., 2015 |
| 20 | There is a lack of complete information about the costs and effectiveness of projects that restore or manage habitats for coastal protection. | LNbS | Narayan et al., 2016 |
| 21 | Natural habitats are dynamic and introduce uncertainty that could be a barrier to the wider use of natural habitats in coastal defence planning. | LNbS | Morris et al., 2018 |
| 22 | The more nature-based a solution is, the higher its demand for land. | LNbS | Hartmann et al., 2019 |
| 23 | Protecting all coastal locations through hard defences where currently planned is not likely to be cost-effective, nor financially realistic. | LGD | Climate Change Committee, 2018 |
| 24 | Hard-engineered structures can lull communities into thinking they are safe from all disasters leading to increased loss of life or property. | LGD | Morris et al., 2018 |
| 25 | Hard structures simply deflect wave energy to adjacent areas | LGD | Bennington-Castro, 2017 |
| 26 | Grey structures are continuing to be built with little positive improvement in practises or management. | LGD | Morris et al., 2018 |
| 27 | Seawalls can reduce the attractiveness of the landscape | LGD | ClimateADAPT, 2016 |
| 28 | Seawalls can destroy natural habitats such as intertidal beaches and dune systems | LGD | Gallop, 2017 |
| 29 | Managed realignment requires significant up-front costs and long-term planning and community engagement. | LMR | Climate Change Committee, 2018 |
| 30 | The process of implementing managed realignment policy can result in social inequalities. | LMR | Environment Agency, 2021 |
| 31 | Communities are likely to be surprised and angered by coastal adaptation policies that do not ‘hold the line’ on existing defences. | LMR | Buser, 2020 |
| 32 | Managed realignment can result in the loss of land area which provides livelihoods to farmers. | LMR | Liski et al., 2019 |
| 33 | A combination of green and grey infrastructures can significantly reduce flooding | BHS | Pamungkas and Purwitaningsih 2019 |
| 34 | Hybrid solutions can be used in areas where there is little space to implement natural approaches alone. | BHS | Sutton Grier et al., 2015 |
| 35 | Within urban environments, hybrid solutions can support resilience to climate change. | BHS | Moosavi, 2017 |
| 36 | Modified seawalls and other hybrid structures are often resource and energy-intensive and lack the capacity to adapt to sea level rise. | LHS | Moosavi, 2017 |
| 37 | Hybrid systems, due to the built part of them, can still have some negative impacts on species diversity. | LHS | Sutton Grier et al., 2015 |
| 38 | Politicians prefer short-term results compared to long-term ones due to the political cycle and the effort to be re-elected. | STPD | Hartmann et al., 2019. Climate Change Committee, 2018 |
| 39 | Long-term strategies are needed to facilitate cost-effective and rapid implementation of integrated ﬂood management. | STPD | Sörensen et al., 2016 |
| 40 | Most adaptation is reactive rather than proactive with the lack of consideration of climate change impacts in coastal planning. | STPD | Masselink et al., 2020 |
| 41 | Risk management authorities can help ensure that natural environment contributes to improving flood and coastal resilience by working closely with those creating/restoring natural habitats. | SE | Environment Agency, 2020 |
| 42 | Stakeholders often tend to fall back on aﬀordable and familiar practices which are less risky and more predictable in their outcomes. | SE | Chee et al., 2021 |
| 43 | Coastal communities need to be engaged to plan for their future over several decades, but the capacity and political will to do so does not currently exist. | SE | Climate Change Committee, 2018 |
| 44 | Public debate should be increased on the possibility and potential need for future relocation of properties and communities. | SE | van der Plank, 2019 |

Table S2. Perspective characteristics

|  | Perspective 1 | Perspective 2 | Perspective 3 | Perspective 4 | Perspective 5 |
| --- | --- | --- | --- | --- | --- |
| Explained variance % | 14 | 8 | 11 | 5 | 8 |
| Eigenvalue | 8.6665 | 1.7071 | 1.4279 | 1.2307 | 1.2328 |
| No. of Defining Variables | 7 | 3 | 4 | 3 | 3 |
| Avg. Rel. Coef. | 0.8 | 0.8 | 0.8 | 0.8 | 0.8 |
| Composite Reliability | 0.966 | 0.923 | 0.941 | 0.923 | 0.923 |
| S.E. of Factor Z-scores | 0.184 | 0.277 | 0.243 | 0.277 | 0.277 |

Table S3. Z-scores for each factor for all participants identified by the sector they represented.

| Participant No | Sector | Factor 1 | Factor 2 | Factor 3 | Factor 4 | Factor 5 |
| --- | --- | --- | --- | --- | --- | --- |
| *Perspective 1* | | | | | | |
| Participant 28 | Charity ^a^ * (Scotland) | 0.4787 | 0.0557 | 0.1625 | 0.038 | 0.1355 |
| Participant 6 | Government agency * (Scotland) | 0.7331 | 0.0464 | 0.1259 | 0.0062 | 0.1827 |
| Participant 1 | Government agency * (Wales) | 0.3597 | 0.2956 | 0.0527 | 0.1407 | 0.0689 |
| Participant 10 | Coastal partnership * (England) | 0.395 | 0.0364 | 0.3073 | 0.2134 | 0.1501 |
| Participant 3 | Government agency (England) | 0.3357 | 0.2876 | 0.3898 | -0.1029 | 0.4331 |
| Participant 29 | Charity * (Scotland) | 0.1445 | 0.0666 | 0.1988 | 0.0832 | 0.3891 |
| Participant 20 | Academia (Scotland) | 0.3007 | 0.4599 | -0.0143 | -0.0139 | 0.2412 |
| Participant 4 | Government agency (Scotland) | 0.2736 | 0.1851 | 0.2943 | 0.1249 | 0.3268 |
| Participant 17 | Consultancy * (England) | 0.3747 | -0.0202 | 0.1985 | 0.186 | -0.0541 |
| Participant 2 | Government agency (England) | 0.0874 | 0.3654 | 0.0513 | 0.2583 | 0.0582 |
| Participant 16 | Consultancy * (Scotland) | 0.5339 | 0.2211 | 0.2458 | 0.0914 | 0.032 |
| *Perspective 2* | | | | | | |
| Participant 18 | Academia * (England) | 0.0704 | 0.591 | 0.2838 | 0.0095 | 0.2362 |
| Participant 13 | Local authority * (Scotland) | 0.0874 | 0.548 | 0.0403 | 0.0359 | -0.2103 |
| Participant 5 | Government agency (Scotland) | 0.1445 | 0.4845 | 0.0338 | 0.037 | 0.4632 |
| Participant 7 | Government agency ^b^ * (England) | 0.3513 | 0.4648 | 0.0075 | -0.1289 | 0.2336 |
| Participant 24 | Resident (England) | -0.1855 | 0.286 | 0.1831 | 0.1611 | -0.0462 |
| *Perspective 3* | | | | | | |
| Participant 21 | Academia * (England) | 0.0429 | 0.3272 | 0.7604 | 0.1081 | 0.0921 |
| Participant 26 | Resident * (England) | 0.248 | -0.0998 | 0.7151 | 0.2359 | 0.0454 |
| Participant 11 | Coastal partnership (Scotland) | 0.3357 | 0.1123 | 0.595 | 0.2085 | 0.4649 |
| Participant 9 | Coastal partnership * (Scotland) | 0.3597 | 0.0595 | 0.5834 | 0.2071 | 0.2244 |
| Participant 15 | Local authority * (Scotland) | 0.3911 | 0.1115 | 0.5483 | -0.1434 | 0.0531 |
| Participant 27 | Charity (England) ^a^ | 0.3007 | 0.3943 | 0.4378 | -0.096 | 0.2523 |
| Participant 25 | Resident (England) | 0.0245 | 0.3029 | 0.3144 | 0.2573 | 0.0885 |
| *Perspective 4* | | | | | | |
| Participant 14 | Local authority * (Scotland) | 0.111 | 0.068 | 0.0361 | 0.6656 | -0.0176 |
| Participant 8 | Coastal partnership * (England) | 0.3467 | 0.0835 | 0.2846 | 0.519 | 0.1601 |
| Participant 12 | Local authority * (England) | 0.2736 | -0.0002 | 0.2819 | 0.5013 | 0.2533 |
| Participant 19 | Academia (England) | 0.1605 | 0.3049 | 0.0027 | -0.3589 | -0.0599 |
| Participant 31 | Charity (England) ^a^ | 0.1678 | 0.2536 | 0.053 | 0.2616 | 0.0295 |
| *Perspective 5* | | | | | | |
| Participant 30 | Charity * (Scotland) | 0.1176 | 0.0346 | 0.1974 | -0.0163 | 0.6273 |
| Participant 23 | Resident * (Scotland) | 0.0413 | -0.0726 | -0.0099 | 0.1418 | 0.6151 |
| Participant 22 | Resident * (Scotland) | 0.3747 | 0.2652 | 0.2513 | 0.0849 | 0.5395 |

*These participants significantly loaded on to just one factor.

^a^ These charities are also involved in land management and thus could be described as land managers.

^b^ This government agency has a different remit, therefore is not included under the risk management authorities.

Table S4. Distinguishing Statements for Factor 1. Q-SV= Idealised Q-sort value. Asterisk (*) Indicates Significance at p<0.01)

| No | Statement | Factor 1 Q-SV | Factor 1 Z-score |
| --- | --- | --- | --- |
| 4 | Nature-based solutions may provide carbon storage and biodiversity improvements | 5 | 1.67 |
| 2 | Nature-based solutions offer a wide assortment of social benefits and community interests such as recreation and wellbeing. | 5 | 1.65 |
| 37 | Hybrid systems, due to the built part of them, can still have some negative impacts on species diversity. | 2* | 0.88 |
| 26 | Grey structures are continuing to be built with little positive improvement in practises or management. | 2* | 0.47 |
| 5 | Taking proactive steps to create nature-based solutions now will save money in the future and help to create a coastline that is naturally resilient to future changes. | 1 | 0.37 |
| 6 | Public opinion currently supports installing, maintaining and funding hard-engineered flood protection structures. | 0 | 0.1 |
| 24 | Hard-engineered structures can lull communities into thinking they are safe from all disasters leading to increased loss of life or property. | -2 | -0.67 |
| 38 | Politicians prefer short-term results compared to long-term ones due to the political cycle and the effort to be re-elected. | -2 | -0.87 |

Table S5. Distinguishing Statements for Factor 2. Q-SV= Idealised Q-sort value. Asterisk (*) Indicates Significance at p<0.01)

| No | Statement | Factor 2 Q-SV | Factor 2 Z-score |
| --- | --- | --- | --- |
| 28 | Seawalls can destroy natural habitats such as intertidal beaches and dune systems | 3 | 1.28 |
| 31 | Communities are likely to be surprised and angered by coastal adaptation policies that do not "hold the line" on existing defences. | 3 | 1.28 |
| 6 | Public opinion currently supports installing, maintaining and funding hard-engineered flood protection structures. | 2 | 0.95 |
| 30 | The process of implementing managed realignment policy can result in social inequalities. | 2 | 0.59 |
| 36 | Modified seawalls and other hybrid structures are often resource and energy-intensive and lack the capacity to adapt to sea level rise. | 1 | 0.56 |
| 4 | Nature-based solutions may provide carbon storage and biodiversity improvements | 0 | -0.14 |
| 2 | Nature-based solutions offer a wide assortment of social benefits and community interests such as recreation and wellbeing. | -1 | -0.43 |
| 14 | Managed realignment can enable more outdoor activities in nature, which could contribute to resident wellbeing. | -1 | -0.44 |
| 32 | Managed realignment can result in the loss of land area which provides livelihoods to farmers. | -5 | -1.75 |

Table S6. Distinguishing Statements for Factor 3. Q-SV= Idealised Q-sort value. Asterisk (*) Indicates Significance at p<0.01)

| No | Statement | Factor 3 Q-SV | Factor 3 Z-score |
| --- | --- | --- | --- |
| 35 | Within urban environments, hybrid solutions can support resilience to climate change. | 3 | 1.01 |
| 11 | Managed realignment can help avoiding scenarios which necessitate long term financial commitment | 3 | 0.99 |
| 19 | Permitting for natural projects can be a more difficult process than for built projects. | 2 | 0.56 |
| 23 | Protecting all coastal locations through hard defences where currently planned is not likely to be cost-effective, nor financially realistic. | -1 | -0.35 |
| 28 | Seawalls can destroy natural habitats such as intertidal beaches and dune systems | -2 | -0.57 |
| 27 | Seawalls can reduce the attractiveness of the landscape | -3 | -1.11 |
| 21 | Natural habitats are dynamic and introduce uncertainty that could be a barrier to the wider use of natural habitats in coastal defence planning. | -4 | -1.68 |

Table S7. Distinguishing Statements for Factor 4. Q-SV= Idealised Q-sort value. Asterisk (*) Indicates Significance at p<0.01)

| No | Statement | Factor 4 Q-SV | Factor 4 Z-score |
| --- | --- | --- | --- |
| 4 | Nature-based solutions may provide carbon storage and biodiversity improvements | 5 | 2.4 |
| 19 | Permitting for natural projects can be a more difficult process than for built projects. | 4 | 1.32 |
| 8 | If designed appropriately, seawalls can increase local ecosystems and biodiversity's resiliency. | 4 | 1.32 |
| 18 | In the case of restored ecosystems, it can take a long time for ecosystems to get established for the natural systems to provide the necessary level of coastal protection. | 2 | 0.62 |
| 13 | Managed realignment could be the only viable option in the long term for some coastal areas. | -3 | -1.15 |
| 29 | Managed realignment requires significant up-front costs and long term planning and community engagement. | -4 | -1.48 |
| 31 | Communities are likely to be surprised and angered by coastal adaptation policies that do not "hold the line" on existing defences. | -4 | -1.66 |

Table S8. Distinguishing Statements for Factor 5. Q-SV= Idealised Q-sort value. Asterisk (*) Indicates Significance at p<0.01)

| No | Statement | Factor 5 Q-SV | Factor 5 Z-score |
| --- | --- | --- | --- |
| 40 | Most adaptation is reactive rather than proactive with the lack of consideration of climate change impacts in coastal planning. | 5 | 1.8 |
| 27 | Seawalls can reduce the attractiveness of the landscape | 4 | 1.37 |
| 25 | Hard structures simply deflect wave energy to adjacent areas | 3 | 1.07 |
| 32 | Managed realignment can result in the loss of land area which provides livelihoods to farmers. | 2 | 0.74 |
| 15 | Managed realignment has the potential to deliver multiple co-benefits. | 0 | -0.16 |
| 21 | Natural habitats are dynamic and introduce uncertainty that could be a barrier to the wider use of natural habitats in coastal defence planning. | -3 | -0.84 |
| 10 | The high level of security provided by a seawall can favour the development of the areas further inland | -5 | -2.24 |

References:

**Bennington-Castro J** (2017) Walls Won't Save Our Cities From Rising Seas. Here's What Will. NBC News. Available online: <https://www.nbcnews.com/mach/science/walls-won-t-save-our-cities-rising-seas-here-s-ncna786811> [Accessed 19 April 2023].

**Buser M** (2020) Coastal Adaptation Planning in Fairbourne, Wales: lessons for Climate Change Adaptation. *Planning Practice & Research*, **35**(2), 127-147. <https://doi.org/10.1080/02697459.2019.1696145>.

**Chee SY, Firth LB, Then AY-H, Yee JC, Mujahid A, Affendi YA, Amir AA, Lau CM, Ooi JLS, Quek YA, Tan CE, Yap TK, Yeap CA, and McQuatters-Gollop A** (2021) Enhancing Uptake of Nature-Based Solutions for Informing Coastal Sustainable Development Policy and Planning: A Malaysia Case Study. *Frontiers in Ecology and Evolution*, **9**. <https://doi.org/10.3389/fevo.2021.708507>.

**ClimateADAPT** (2016) Seawalls and jetties. Available online: <https://climate-adapt.eea.europa.eu/en/metadata/adaptation-options/seawalls-and-jetties> [Accessed 19 April 2023].

**Committee on Climate Change** (2018) Managing Coast in Changing Climate.

**Doelle M and Puthucherril TG** (2021) Nature‐based solutions to sea level rise and other climate change impacts on oceanic and coastal environments: a law and policy perspective. *Nordic Journal of Botany*. <https://doi.org/10.1111/njb.03051>.

**Environment Agency** (2020) National Flood and Coastal Erosion Risk Management Strategy for England. ISBN 978-1-5286-1791-8.

**Environment Agency** (2021) Supporting flood and coastal erosion risk management through partnerships. Key lessons. Report: FRS17186/2.

**Gallop SL** (2017) Are seawalls the magic solution to coastal flooding and erosion? SurgeWatch. Available online: <https://www.surgewatch.org/are-seawalls-the-magic-solution-to-coastal-flooding-and-erosion/> [Accessed 19 April 2023].

**Hartmann T, Slavíková L and McCarthy S** (2019) Nature-Based Flood Risk Management on Private Land. Disciplinary Perspectives on a Multidisciplinary Challenge. Springer, pp. 224. <https://doi.org/10.1007/978-3-030-23842-1>.

**Liski AH, Ambros P, Metzger MJ, Nicholas KA, Wilson AMW and Krause T** (2019) Governance and stakeholder perspectives of managed re-alignment: adapting to sea level rise in the Inner Forth estuary, Scotland. *Regional Environmental Change*, **19**(8), 2231-2243. <https://doi.org/10.1007/s10113-019-01505-8>.

**MacDonald MA, de Ruyck C, Field RH, Bedford A and Bradbury RB** (2017) Benefits of coastal managed realignment for society: Evidence from ecosystem service assessments in two UK regions. *Estuarine, Coastal and Shelf Science*, **244**, 105609. <https://doi.org/10.1016/j.ecss.2017.09.007>.

**Masselink G, Russell P, Rennie A, Brooks S and Spencer T** (2020) Impacts of climate change on coastal geomorphology and coastal erosion relevant to the coastal and marine environment around the UK. *MCCIP Science Review*, 158-189. doi: 10.14465/2020.arc08.cgm.

**Moosavi S** (2017) Ecological Coastal Protection: Pathways to Living Shorelines. *Procedia Engineering*, **196**, 930-938. <https://doi.org/10.1016/j.proeng.2017.08.027>.

**Morris RL, Konlechner TM, Ghisalberti M and Swearer SE** (2018) From grey to green: Efficacy of eco-engineering solutions for nature-based coastal defence. *Global Change Biology*, **24**(5), 1827-1842. <https://doi.org/10.1111/gcb.14063>.

**Narayan S, Beck MW, Reguero BG, Losada IJ, van Wesenbeeck B, Pontee N, Sanchirico JN, Ingram JC, Lange GM and Burks-Copes KA** (2016) The Effectiveness, Costs and Coastal Protection Benefits of Natural and Nature-Based Defences. *PLoS One*, **11**(5), e0154735. <https://doi.org/10.1371/journal.pone.0154735>.

**Pamungkas A and Purwitaningsih S** (2019) Green and grey infrastructures approaches in flood reduction. *International journal of disaster resilience in the built environment* **10** (5), 343-362. <https://doi.org/10.1108/IJDRBE-03-2019-0010>.

**Rendon OR, Sandorf ED and Beaumont NJ** (2022) Heterogeneity of values for coastal flood risk management with nature-based solutions. Journal of Environmental Management, **304**, 114212. <https://doi.org/10.1016/j.jenvman.2021.114212>.

**Rennie AF, Hansom JD, Hurst MD, Muir FME, Naylor LA, Dunkley RA and MacDonell CJ** (2021) Dynamic Coast. The National Overview. CRW2017_08. Scotland's Centre of Expertise for Waters (CREW). Available online at: crew.ac.uk/publications.

**Sörensen J, Persson A, Sternudd C, Aspegren H, Nilsson J, Nordström J, Jönsson K, Mottaghi M, Becker P, Pilesjö P, Larsson R, Berndtsson R and Mobini S** (2016) Re-Thinking Urban Flood Management—Time for a Regime Shift. *Water*, **8**(8), 332. <https://doi.org/10.3390/w8080332>.

**Sutton-Grier AE, Wowk K and Bamford H** (2015) Future of our coasts: The potential for natural and hybrid infrastructure to enhance the resilience of our coastal communities, economies and ecosystems. *Environmental Science & Policy*, **51**, 137-148. <https://doi.org/10.1016/j.envsci.2015.04.006>.

**Van Der Plank S, Brown S, Nicholls RJ and Tompkins EL** (2020) Stakeholder expectations of the public in local coastal flood risk management in England. In Coastal Management 2019: Joining forces to shape our future coasts (pp. 605-618). ICE Publishing. <https://doi.org/10.1680/cm.65147.605>.

**Welsh Government** (2020) The National Strategy for Flood and Coastal Erosion Risk Management in Wales. ISBN 978-1-80082-342-6.

**Williams AT, Rangel-Buitrago N, Pranzini and Anfuso G** (2018) The management of coastal erosion. *Ocean & Coastal Management*, **156**, 4-20. <https://doi.org/10.1016/j.ocecoaman.2017.03.022>.
