# Supplementary material for: Is the coastal future green, grey or hybrid? Diverse perspectives on coastal flood risk management and adaptation in the UK
Source: Camb Prism Coast Futur. 2024 Feb 12;2:e4. doi: 10.1017/cft.2024.4 (PMC12337589; doi:10.1017/cft.2024.4)
Supplement: Peer review history

# Editor report: Recommendation: Is the coastal future green, grey or hybrid? Diverse perspectives on coastal flood risk management and adaptation in the UK — R0/PR2

**DOI:** 10.1017/cft.2024.4.pr2

**Affiliations:** University of Cantabria, Spain

Dear Dr. Apine,

Regarding your manuscript submitted to Coastal Futures“Is the coastal future green, grey or hybrid? Diverse perspectives on coastal flood risk management and adaptation in the UK”, based on the examination of the reviews, your manuscript requires Major Revision.

Sincerely,

Dr. Fernando Mendez

Cambridge Prisms: Coastal Futures

# Editor report: Decision: Is the coastal future green, grey or hybrid? Diverse perspectives on coastal flood risk management and adaptation in the UK — R0/PR3

**DOI:** 10.1017/cft.2024.4.pr3

**Affiliations:** Geography, Cambridge University, United Kingdom of Great Britain and Northern Ireland

No accompanying comment.

# Editor report: Recommendation: Is the coastal future green, grey or hybrid? Diverse perspectives on coastal flood risk management and adaptation in the UK — R1/PR5

**DOI:** 10.1017/cft.2024.4.pr5

**Affiliations:** University of Cantabria, Spain

No accompanying comment.

# Editor report: Decision: Is the coastal future green, grey or hybrid? Diverse perspectives on coastal flood risk management and adaptation in the UK — R1/PR6

**DOI:** 10.1017/cft.2024.4.pr6

**Affiliations:** Geography, Cambridge University, United Kingdom of Great Britain and Northern Ireland

No accompanying comment.

# Editor report: Recommendation: Is the coastal future green, grey or hybrid? Diverse perspectives on coastal flood risk management and adaptation in the UK — R2/PR8

**DOI:** 10.1017/cft.2024.4.pr8

**Affiliations:** Geography, Cambridge University, United Kingdom of Great Britain and Northern Ireland

The minor revisions undertaken now make this paper acceptable for publication.

# Editor report: Decision: Is the coastal future green, grey or hybrid? Diverse perspectives on coastal flood risk management and adaptation in the UK — R2/PR9

**DOI:** 10.1017/cft.2024.4.pr9

**Affiliations:** Geography, Cambridge University, United Kingdom of Great Britain and Northern Ireland

No accompanying comment.
